# Varied effects of tobacco smoke and e-cigarette vapor suggest that nicotine does not affect endothelium-dependent relaxation and nitric oxide signaling

**DOI:** 10.1038/s41598-023-42750-6

**Published:** 2023-09-22

**Authors:** Gerald Wölkart, Alexander Kollau, Michael Russwurm, Doris Koesling, Astrid Schrammel, Bernd Mayer

**Affiliations:** 1 Department of Pharmacology and Toxicology, Institute of Pharmaceutical Sciences, Universität Graz, Humboldtstraße 46, 8010 Graz, Austria; 2 Department of Pharmacology and Toxicology, Ruhr-Universität Bochum, MA N1-39, 44780 Bochum, Germany

**Keywords:** Preclinical research, Cardiovascular biology, Lifestyle modification

## Abstract

Chronic smoking causes dysfunction of vascular endothelial cells, evident as a reduction of flow-mediated dilation in smokers, but the role of nicotine is still controversial. Given the increasing use of e-cigarettes and other nicotine products, it appears essential to clarify this issue. We studied extracts from cigarette smoke (CSE) and vapor from e-cigarettes (EVE) and heated tobacco (HTE) for their effects on vascular relaxation, endothelial nitric oxide signaling, and the activity of soluble guanylyl cyclase. The average nicotine concentrations of CSE, EVE, and HTE were 164, 800, and 85 µM, respectively. At a dilution of 1:3, CSE almost entirely inhibited the relaxation of rat aortas and porcine coronary arteries to acetylcholine and bradykinin, respectively, while undiluted EVE, with a 15-fold higher nicotine concentration, had no significant effect. With about 50% inhibition at 1:2 dilution, the effect of HTE was between CSE and EVE. Neither extract affected endothelium-independent relaxation to an NO donor. At the dilutions tested, CSE was not toxic to cultured endothelial cells but, in contrast to EVE, impaired NO signaling and inhibited NO stimulation of soluble guanylyl cyclase. Our results demonstrate that nicotine does not mediate the impaired endothelium-dependent vascular relaxation caused by smoking.

## Introduction

Cigarette smoking is an established risk factor for developing severe cardiovascular diseases, including atherosclerosis, coronary artery disease, and stroke^1,2^. The impaired function of vascular endothelial cells exposed to toxic compounds in tobacco smoke contributes significantly to the cardiovascular harm of smoking^3^. Vascular endothelial cells exert various protective effects, and dysfunction of these cells results in inflammation, activation of blood platelets, and vasoconstriction. Endogenous receptor agonists, such as acetylcholine, histamine or bradykinin, as well as increased blood flow trigger endothelium-dependent relaxation of blood vessels through stimulation of endothelial nitric oxide (NO) synthase (eNOS), resulting in NO-mediated activation of soluble guanylyl cyclase (sGC) and accumulation of 3′,5′-cyclic GMP (cGMP) in adjacent vascular smooth muscle cells^4^. Endothelium-dependent vasodilation of the brachial artery in response to increased blood flow, provoked by a short period of ischemia, can be measured noninvasively by ultrasound in laboratory animals and humans^5^. Using this flow-mediated dilation (FMD) approach and other techniques, numerous clinical studies provided evidence for pronounced endothelial dysfunction of chronic smokers^3^. It is conceivable that oxidative stress caused by smoking reduces the bioavailability of endothelium-derived NO through increased generation of superoxide and peroxynitrite formation^6^ or oxidation of the eNOS cofactor tetrahydrobiopterin^7,8^.

In the past decade, new nicotine products have become available, which allow users nicotine consumption without inhaling the smoke of burned tobacco. Electronic cigarettes (e-cigarettes) vaporize nicotine-containing liquids to generate aerosols with liquid droplets (“vapor”) that are inhaled by users^9^. While e-cigarettes do not contain tobacco, tobacco heating systems (THS) generate aerosols by heating tobacco sticks to a maximum 300 °C, a temperature below the threshold for combustion^10^. Since these alternative products do not generate toxic combustion products, users are exposed to lower levels of potentially hazardous substances than smokers^11,12^. Given the large body of evidence demonstrating reduced risks, these alternative nicotine products are recommended by health organizations as alternatives to smokers who are unable or unwilling to quit^13^.

Despite unequivocal evidence for their potential to reduce the harm of smoking, these new products are not entirely risk-free, and there is an ongoing debate about the regulatory measures required to protect adolescents and non-smokers from risks posed by e-cigarettes and THS. Moreover, besides referring to the potentially harmful effects of flavoring compounds, oxidation products of solvents formed in liquid vaporization, or toxicants extracted from heated tobacco, it has been emphasized that most smokers switching to these products will still consume comparable amounts of nicotine as they did before. Thus, sustained nicotine addiction and cardiovascular harm could still be an issue. In addition, although clinical studies showed that nicotine administered via patches or other medicinal products does not significantly increase the risk for severe cardiovascular disease^14,15^, a residual risk of nicotine inhalation with e-cigarettes or THS cannot be excluded^16^.

Considering the consistent results showing endothelial dysfunction of chronic smokers (for review, see Golbidi et al.^3^), clarifying whether nicotine contributes to this harmful effect of tobacco smoke appears essential. Several recent studies have addressed this issue using different experimental approaches, including FMD measurements of humans. Unfortunately, these studies did not provide consistent results. While some studies reported reduced FMD^17–20^ or impaired microvascular funtion^21^ by acute inhalation of nicotine-containing e-cigarette vapor, others observed no acute effects of nicotine inhalation^22,23^. Several studies have recently addressed the mid-term and long-term changes of FMD in smokers who replaced tobacco cigarettes with e-cigarettes with and without nicotine. George et al.^24^ showed that the endothelial function of smokers was significantly improved one month after replacing tobacco cigarettes by e-cigarettes with or without nicotine. Similar results were reported by Klonizakis et al*.*^25^, showing that the reduction of FMD persisted for at least six months in smokers who had either received nicotine replacement therapy or switched to e-cigarettes with or without nicotine. These observations were recently confirmed by the *VAPing Observational Research Study-Endothelial Function* (VAPORS-E) study data. Individuals who had exclusively used e-cigarettes for at least six months, the majority with nicotine according to cotinine plasma levels, did not differ significantly from non-users in their mean FMD^26^. Taken together, these results suggest that the chronic endothelial dysfunction of smokers is not caused by nicotine but by toxic constituents in the smoke of burned tobacco. However, Mohammadi et al. reported that chronic e-cigarette users and smokers had lower FMD than non-users^27^, indicating that this issue has not yet been fully settled.

The results reported with laboratory animals, isolated blood vessels, and cultured endothelial cells exposed to smoke or vapor are also controversial. While tobacco smoke extracts were consistently reported to cause vascular and endothelial dysfunction, presumably via increased oxidative stress^28–30^, the role of nicotine is less clear. Some studies reported impaired endothelial function in mice and rats exposed to nicotine-containing e-cigarette vapor^31–33^. In contrast to these findings, activating nicotinic receptors appears to cause NO release in the brain^34^, in renal blood vessels^35^, and in several types of human vascular endothelial cells^36,37^. In line with these observations, Zou et al.^38^ found that activation of nicotinic receptors contributes to acetylcholine-induced relaxation of rat aorta, and Mizrak et al*.*^39^ reported that the vasorelaxation to acetylcholine was significantly enhanced in rats fed for 12 months with nicotine in drinking water.

In the present study, we compared the effects of aqueous smoke extracts from Marlboro cigarettes (cigarette smoke extracts, CSE) with extracts of vapor generated by a popular e-cigarette device filled with a nicotine-containing liquid (20 mg/ml) (e-cigarette vapor extracts, EVE) or the aerosol emitted by a THS (heated tobacco extracts, HTE) on endothelium-dependent and -independent relaxation of rat aorta ex vivo. In addition, we studied NO/cGMP signaling and cytotoxicity in cultured endothelial cells and measured the NO-dependent and -independent activities of purified sGC in the presence of aerosol extracts. The potential scavenging of NO by the extracts was studied with a NO-sensitive electrode. We observed pronounced differences between CSE and EVE or HTE that did not correlate with measured nicotine concentrations, suggesting that nicotine does not significantly impair endothelial function ex vivo and in vitro.

## Methods

### Animals and tissue preparation

All animal experiments were performed in compliance with the Austrian law on experimentation with laboratory animals (last amendment 2013) based on the European Union guidelines for the Care and the Use of Laboratory Animals (European Union Directive 2010/63/EU) and are reported in accordance with the ARRIVE guidelines for reporting experiments involving animals^40^. The killing of animals solely for the use of their organs or tissues is explicitly excluded in Article 3 (page L276/39) of EU Directive 2010/63/EU on the protection of animals used for scientific purposes.

Sprague–Dawley rats (total number 15) of either sex were obtained from Charles River Laboratories (Sulzfeld, Germany) and housed at the local animal facility until they were used for the study at 16–20 weeks of age (body weight: 250–300 g). Rats were kept in approved conventional polycarbonate cages (Ehret, Emmerdingen, Germany), two to three animals per cage with dust-free laboratory bedding and enrichment (nesting material and rodent tunnels; from Abedd GmbH, Vienna, Austria). Animals were maintained at 23 ± 1 °C, with a relative humidity of 50–70% and kept on a regular 12-h dark/light cycle. Housing and care of the animals was according to the guidelines of the Austrian Federal Ministry of Science and Research and is periodically controlled by the authority. Rats were fed standard chow (Altromin 3023; obtained from Königshofer Futtermittel (Ebergassing, Austria)) and received water ad libitum. The animals were euthanized in a box gradually filled with CO_2_ until no more vital signs (cessation of respiration and circulation) were noticed. Subsequently, the thorax of the animals was opened, the thoracic aorta removed, cleaned from connective tissue, and used for assessment of vessel function. Aortas were randomly assigned to the different treatment protocols, with none excluded.

Porcine hearts were obtained from a local abattoir and immediately transported to the laboratory. The right coronary artery was carefully explanted, cleaned from connective tissue, and used to assess vessel function.

### Preparation of aqueous extracts from tobacco cigarettes, THS and e-cigarettes

According to a published protocol^41^, aqueous extracts were obtained by passing cigarette smoke, e-cigarette vapor, or THS aerosol through 30 ml Dulbecco's Modified Eagle’s Medium (DMEM) supplemented with amphotericin B (1.25 µg/ml), penicillin (100 U/ml) and streptomycin (0.1 µg/ml) using a glass impinger. A rodent ventilator (Ugo Basile, Italy) connected to the glass impinger acted as a puffing machine to ensure a constant puffing regime (55 ml puff taken over 3 s in 30-s intervals, square wave puffing profile). For adequate comparison of the effects of the extracts, stroke rates, volume, and the length of tubing was kept consistent for all experimental protocols.

Based on recent sales data, showing that Marlboro is still the most popular cigarette brand^42^, commercially available Marlboro red cigarettes (0.8 mg nicotine, 10 mg tar, Philip Morris) were used to prepare CSE. Aqueous extracts were generated by passing 800 ml cigarette smoke through 30 ml DMEM.

The same setup was used to prepare aqueous EVE using an Eleaf iStick 50 W battery with Aspire Nautilus X 2 ml tanks and Nautilus X atomizer heads (1.8 Ohm; InnoCigs GmbH & Co KG, Germany) operated at 15.0 W. The battery was fully charged before use, and the tank was filled with pure unflavored e-liquid (i-steam; ROPA, Vienna, Austria) containing nicotine (20 mg/ml) in 50/50 (vol/vol.) glycerol/propylene glycol. E-cigarettes were activated by manually holding a button for 5 s in 5-s intervals. In order to achieve the same conditions as for preparing cigarette extracts, 800 ml of EVE was passed through 30 ml DMEM.

HTE was prepared with an IQOS 3 DUO (Philip Morris), equipped with commercially available tobacco sticks ("Heets", Siena Selection, red label, 0.5 mg nicotine; Philip Morris). To achieve the same vaping time and aerosol volume (800 ml) as for cigarettes and e-cigarettes, two Heets were used to prepare one extract. Passing cigarette smoke, e-cigarette vapor or THS aerosol through DMEM did not significantly affect pH values (pH 7.4 ± 0.2).

For HPLC analysis, sGC activity assays, and electrochemical NO measurements, the extracts were prepared in 50 mM triethanolamine buffer (TEA/HCl, pH 7.4), as DMEM interfered with these methods. For cell culture experiments, extracts were passed through sterile syringe filters (pore diameter 0.2 µm).

### Determination of nicotine

The nicotine concentration of the extracts was determined using a 1260 Infinity HPLC System from Agilent equipped with a diode-array detector using a reversed-phase poroshell 120 EC-C18 column (4.6 × 50 mm, 2.7 µm). Elution was carried out isocratically using 70% of 20 mM phosphate buffer (pH 7.4), and 30% of methanol.

The extracts were passed through a syringe filter with 0.2 µm pore size, 10 µl aliquots were injected onto the column and separated at a flow rate of 1.0 ml/min at a column temperature of 25 °C. For quantification, calibration curves with a linear range from 0.003 to 0.3 mg/ml were established with analytical grade nicotine (Sigma, N5511) and analyzed based on the area of the peak detected at 260 nm (retention time 4.0 min).

### Electrochemical determination of NO

Potential NO scavenging by aqueous extracts was tested with a nitric oxide sensor (ISO-NOP, World Precision Instruments, Berlin, Germany) connected to a TBR 4100 Free Radical Analyzer (World Precision Instruments). The sensor was calibrated daily with acidified nitrite as described previously^43^. Free NO radical was generated by incubation of 2-(*N*,*N*-diethylamino)-diazenolate-2-oxide diethylammonium salt (DEA/NO) at the indicated concentrations in 1 ml of 50 mM TEA/HCl buffer (pH 7.4), or the respective extract at 37 °C and monitored using LabChart 6 Software (ADInstruments, Version 6.1.3).

### Isometric tension vasomotor studies

Rings with intact endothelium obtained from one vessel were preincubated in DMEM alone or different CSE, EVE, or HTE dilutions for 24 h at 37 °C and 5% CO_2_. Rings were randomly assigned to the different incubation protocols to avoid any systematic effects due to the repeated use of similar vessel segments. Rings were then suspended in 5-ml organ baths containing oxygenated Krebs–Henseleit buffer (concentrations in mM: NaCl 118.4, NaHCO_3_ 25, KCl 4.7, KH_2_PO_4_ 1.2, CaCl_2_ 2.5, MgCl_2_ 1.2, D-Glucose 11; pH 7.4), as previously described in detail^44^. After equilibration for 60 min at the optimal resting tension of 20 mN, maximal contractility was determined with a depolarizing solution containing 60 mM KCl. Rings that did not elicit adequate and stable contraction to high K^+^ were considered damaged and omitted from the study. Cumulative concentration–response curves to acetylcholine (1 nM–10 µM) or DEA/NO (1 nM–10 µM) were performed with rings that had been pre-contracted with 9,11-dideoxy-9α,11α-epoxy-methanoprostaglandin F_2α_ (U-46619; ~ 90% of maximal contraction). To examine endothelial-dependent relaxation of porcine coronary arteries, concentration–response curves to bradykinin (0.1 nM–1 µM) in high K^+^ precontracted rings were carried out. Relaxation responses are expressed as percent reversal of U-46619 or K^+^-induced precontraction. Concentration–response curves of different ring segments from a single animal were averaged and counted as an individual experiment.

### Cell culture

Porcine aortic endothelial cells (PAECs) were isolated as described^45^ and cultured at 37 °C, 5% CO_2_, and 80% humidity in DMEM containing 10% heat-inactivated fetal calf serum (FCS), penicillin (100 U/ml), streptomycin (0.1 mg/ml), and amphotericin (1.25 μg/ml). Cells were subcultured on 24-well plates to determine cGMP formation or on 6-well plates to analyze endothelial L-citrulline formation.

### Determination of endothelial NOS activity and cGMP accumulation

NO synthase (NOS) activity was determined in PAECs by monitoring the conversion of L-[^3^H]arginine to L-[^3^H]citrulline as described^46^. Cells were preincubated with the extracts diluted in DMEM for 24 h at 37 °C and then assayed in 50 mM Tris buffer (pH 7.4) containing 100 mM NaCl, 5 mM KCl, 1 mM MgCl_2_, and 2.5 mM CaCl_2_. Preincubations were performed in the absence and presence of the non-selective NO synthase inhibitor N^G^-nitro-L-arginine (L-NNA; 1 mM). Reactions were started by the addition of L-[2,3,4,5-^3^H]arginine (~ 10^6^ dpm) and the Ca^2+^-ionophore A23187 (1 μM) and terminated after 4 min by washing the cells with chilled incubation buffer. After lysis of the cells with 10 mM HCl (1 ml) for 1 h at ambient temperature, an aliquot (0.1 ml) was removed to determine incorporated radioactivity. The remaining sample was mixed with 200 mM sodium acetate buffer (pH 13.0) containing 10 mM L-citrulline (final pH ~ 5.0), and L-[^3^H]citrulline was separated from L-[^3^H]arginine by cation exchange chromatography, followed by determination of radioactivity by liquid scintillation counting.

Accumulation of intracellular cGMP was determined as described^44^. Cells were preincubated for 24 h at 37 °C with the extracts diluted in medium and then assayed in 50 mM Tris buffer (pH 7.4), containing 100 mM NaCl, 5 mM KCl, 1 mM MgCl_2_, 2.5 mM CaCl_2_, 1 mM 3-isobutyl-1-methylxanthine, and 1 μM indomethacin. Reactions were started by addition of A23187 or the NO donor DEA/NO (1 μM, each) in the absence and presence of 1 mM L-NNA and terminated after 4 min by removal of incubation medium, followed by cell lysis with 10 mM HCl for 1 h at ambient temperature and determination of released cGMP by radioimmunoassay.

Acute effects of CSE and EVE on eNOS activity were studied by incubating the cells for 15 min in the presence of the extracts under identical conditions with and without 1 mM L-NNA, followed by determination of L-[^3^H]arginine to L-[^3^H]citrulline conversion.

### Determination of endothelial cell viability

Cell viability was assessed using the XTT Cell Proliferation Assay Kit (#10,010,200) from Cayman Chemical, purchased through Sanova Pharma GesmbH (Vienna, Austria). PAECs were seeded on 96-well plates and incubated for 24 h with the extracts diluted in DMEM, as indicated in the text and figure. Colorimetric reactions, based on the reduction of 2,3-bis-(2-methoxy-4-nitro-5-sulfophenyl)-2H-tetrazolium-5-carboxanilide (XTT) to a water-soluble formazan derivative by respiring cells, were carried out according to the manufacturer’s instructions. Absorbance was measured at 450 nm using a SPECTROstar^®^ Nano microplate reader (BMG LABTECH GmbH, Ortenberg, Germany).

### Determination of cGMP formation by purified sGC

Bovine lung sGC was purified as described^47^. The enzyme was incubated at 37 °C for 10 min in a final volume of 0.1 ml in the absence or presence of the extracts diluted as indicated in the figures. The final assay mixtures contained the extracts at 88% (vol/vol). The resulting final nicotine concentrations in undiluted EVE and CSE were 23.8 and 114.4 µg/ml, respectively. Assay mixtures contained 50 mM TEA/HCl buffer (pH 7.4), 0.5 mM [α-^32^P]GTP (∼200,000 cpm), 3 mM MgCl_2_, and 1 mM cGMP. Reactions were started by adding DEA/NO or cinaciguat at the indicated concentrations and terminated by adding 0.45 ml zinc acetate (120 mM) and 0.45 ml sodium bicarbonate (120 mM). After centrifugation (20,000×*g* at 4 °C for 10 min), supernatants were applied onto Al_2_O_3_ columns (acidified with 0.1 M HClO_4_). After washing the columns with distilled water, [^32^P]cGMP was eluted with sodium acetate (50 mM) and quantified by liquid scintillation counting. Blank values were measured in the absence of sGC.

Basal enzyme activity was determined without any stimulating agent (*i.e.,* DEA/NO or cinaciguat). The activity of the oxidized enzyme was determined in the presence of 10 µM of the heme-oxidizing agent 1*H*-[1,2,4]oxadiazolo-[4,3-a]quinoxalin-1-one (ODQ)^48^. Heme-depleted sGC was generated by adding 0.5% (vol./vol.) Tween 20 during incubation^49^. The experiments with the activator cinaciguat were performed in the presence of 0.5% Tween 20.

To test for reversibility of effects caused by the extracts, sGC (50 ng) was preincubated in the absence or presence of the extracts for 5 min at 37 °C and then diluted 35-fold in the assay mixture. Reactions were started by addition of DEA/NO, and cGMP formation was determined as described above.

### Randomization and blinding

Laboratory animals were randomly assigned to experiments. Porcine hearts were collected by slaughters of the abattoir, and the researcher had no influence on the selection of hearts. Experiments were not blinded in this study, as investigators were responsible for conducting and analyzing experiments. Blinding is not a usual procedure for this form of study and cannot be applied retrospectively. To reduce experimental bias, data analysis was not performed until the complete data set had been collected, and no complete recording was excluded.

### Statistical analysis

Results are presented as mean values ± SEM of n experiments. Maximal effects (E_max_ and v_max_) and half-maximal effective concentrations (EC_50_ values) were obtained by fitting concentration–response curves to a Hill-type model using Kaleidagraph (Synergy Software, version 4.5). EC_50_ values are reported as geometric means with 95% confidence limits. Paired Student’s t-test or analysis of variance (ANOVA) with Dunnett's post hoc test was used to compare groups using Kaleidagraph software. Significance was assumed at *p* < 0.05.

## Results

### Nicotine concentration of the aerosol extracts

The nicotine concentrations measured in the aqueous extracts prepared with the aerosols generated by tobacco cigarettes, e-cigarettes, and THS were 26.7 ± 0.9 µg/ml (164 ± 5.4 µM), 129.7 ± 26 µg/ml (800 ± 16 µM), and 13.8 ± 0.4 µg/ml (85 ± 2.3 µM), respectively (Table 1). These values agree well with previously published data obtained with the same protocol^41^. The vaporized volume of e-cigarette liquid (containing 20 mg of nicotine per ml) was 257 ± 0.18 µl (n = 3), corresponding to a theoretical nicotine concentration of 171 µg/ml in EVE (30 ml). Thus, about 25% of nicotine in the e-cigarette liquid was lost during the procedure, possibly due to partial decomposition or escape from the collecting fluid. Note that the nicotine concentrations of the undiluted extracts used in this study are about 1800- (CSE), 900- (HTE), and 4300-fold (EVE) higher than the average plasma concentration of chronic smokers, which is around 30 ng/ml^50^.

**Table 1:** Nicotine concentrations of three separately prepared aqueous extracts were analyzed by HPLC.

Sample	mg/ml	mmol/l
**CSE**		
#1	0.027	0.166
#2	0.028	0.173
#3	0.025	0.154
Mean ± SEM	0.0267 ± 0.0009	0.164 ± 0.005
**EVE**		
#1	0.125	0.77
#2	0.130	0.80
#3	0.134	0.83
Mean ± SEM	0.1297 ± 0.0026	0.799 ± 0.016
**HTE**		
#1	0.015	0.089
#2	0.014	0.084
#3	0.013	0.081
Mean ± SEM	0.0138 ± 0.0004	0.085 ± 0.002

### Effects of aerosol extracts on vascular relaxation

Rings with intact endothelium of rat aorta or porcine coronary arteries were preincubated for 24 h with aqueous extracts diluted as indicated in the figures, followed by recording concentration–response curves to the endothelium-dependent vasodilator acetylcholine or bradykinin, respectively. Endothelium-independent relaxation was induced with the NO donor DEA/NO.

**Figure 1:**
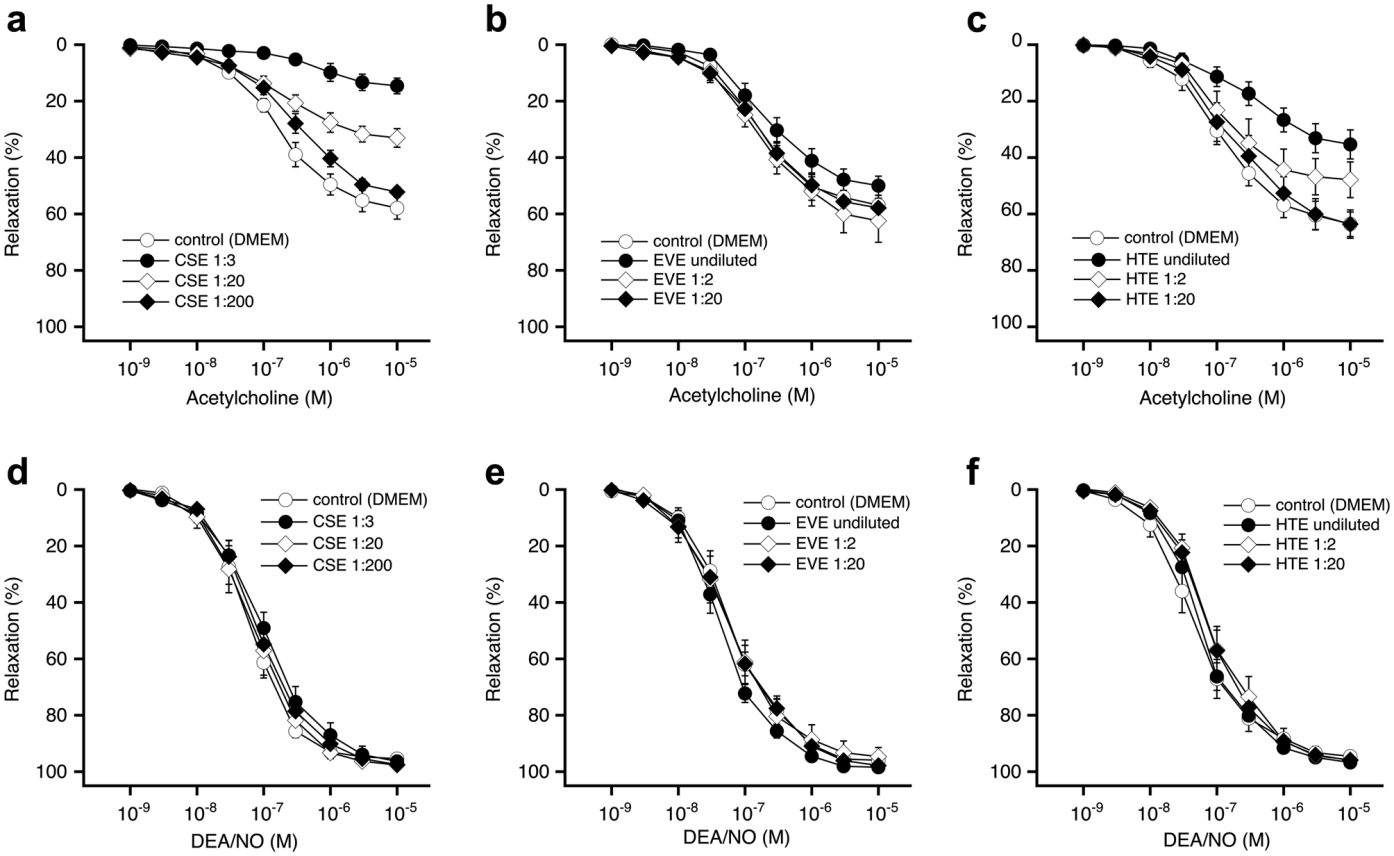
Effects of aerosol extracts on relaxation of rat aortic rings to acetylcholine or DEA/NO. CSE (**a**) and HTE (**c**), but not EVE (**b**) impaired endothelial dependent relaxation. In contrast, endothelium-independent relaxation induced with the NO donor DEA/NO was not affected by CSE (**d**), EVE (**e**) or HTE (**f**). Two rings from a single animal were averaged and counted as an individual experiment. Data are expressed as mean values ± SEM derived from 5 animals. For Statistics see Table 2.

Figure 1a shows that CSE impaired acetylcholine-induced relaxation of rat aorta by about 50% at 1:20 dilution and almost entirely inhibited vasodilation if the vessels had been preincubated with a 1:3 diluted extract. In contrast, even undiluted EVE had only very minor effects on relaxation to acetylcholine (Fig. 1b). HTE inhibited acetylcholine-induced relaxation but to a significantly lesser extent than CSE (Fig. 1c). The vasodilation caused by the NO donor DEA/NO was not affected by any of the extracts (Fig. 1d–f), suggesting that CSE and HTE specifically impair endothelium-dependent relaxation. The effects of the extracts on E_max_ and EC_50_ on relaxation of rat aortas in response to acetylcholine and DEA/NO are summarized in Table 2. While CSE in 1:20 dilution containing 1.35 µg/ml nicotine reduced E_max_ in response to acetylcholine to 58% of controls, undiluted EVE with a 100-fold higher nicotine concentration (130 µg/ml) had no significant effect. The effect of HTE was in between those of CSE and EVE. At 1:20 dilution, corresponding to 0.70 µg/ml nicotine, the extract had no effect but reduced E_max_ by about 50% at lower dilutions. Endothelium-independent vasodilation induced with the NO donor DEA/NO was not affected under any experimental condition. The lack of significant effects on acetylcholine potency (EC_50_ values) suggests non-competitive inhibition of endothelium-dependent relaxation by CSE and—to a lesser extent—by HTE.

**Table 2:** Effects of CSE, EVE, and HTE on relaxation of rat aortas to acetylcholine and DEA/NO.

Treatment	Nicotine µg/ml	Acetylcholine		DEA/NO	
		E_max_ (%)	EC_50_ (nM)	E_max_ (%)	EC_50_ (nM)
**CSE**					
Control	–	59 ± 3.8	170 (94–307)	96 ± 2.1	60 (38–94)
CSE 1:3	6.75	17 ± 3.8*	806 (233–2790)	97 ± 1.7	95 (49–187)
CSE 1:20	1.35	34 ± 3.4*	190 (93–390)	98 ± 1.2	69 (29–168)
CSE 1:200	0.13	56 ± 2.7	335 (144–777)	97 ± 0.9	80 (43–150)
**EVE**					
Control	–	57 ± 5.7	150 (89–251)	96 ± 1.9	63 (28–141)
EVE undil	130	50 ± 4.2	190 (67–537)	98 ± 0.9	44 (26–74)
EVE 1:2	43.3	64 ± 8.1	166 (131–210)	95 ± 3.3	53 (21–135)
EVE 1:20	6.50	60 ± 4.6	180 (89–365)	99 ± 0.5	58 (34 -100)
**HTE**					
Control	–	63 ± 5.0	109 (50–238)	94 ± 1.6	46 (19–112)
THP undil	14	37 ± 5.5*	278 (84–925)	96 ± 1.4	87 (21–362)
THP 1:2	4.67	49 ± 6.2	146 (62–345)	96 ± 1.6	90 (41–201)
THP 1:20	0.70	64 ± 4.7	167 (54–514)	95 ± 1.9	76 (37–159)

Data shown are mean values ± SEM (E_max_) or mean values with 95% confidence interval (EC_50_) from 5 experiments. *p < 0.05 vs. control as determined by ANOVA and Dunnett’s post hoc test.

**Figure 2:**
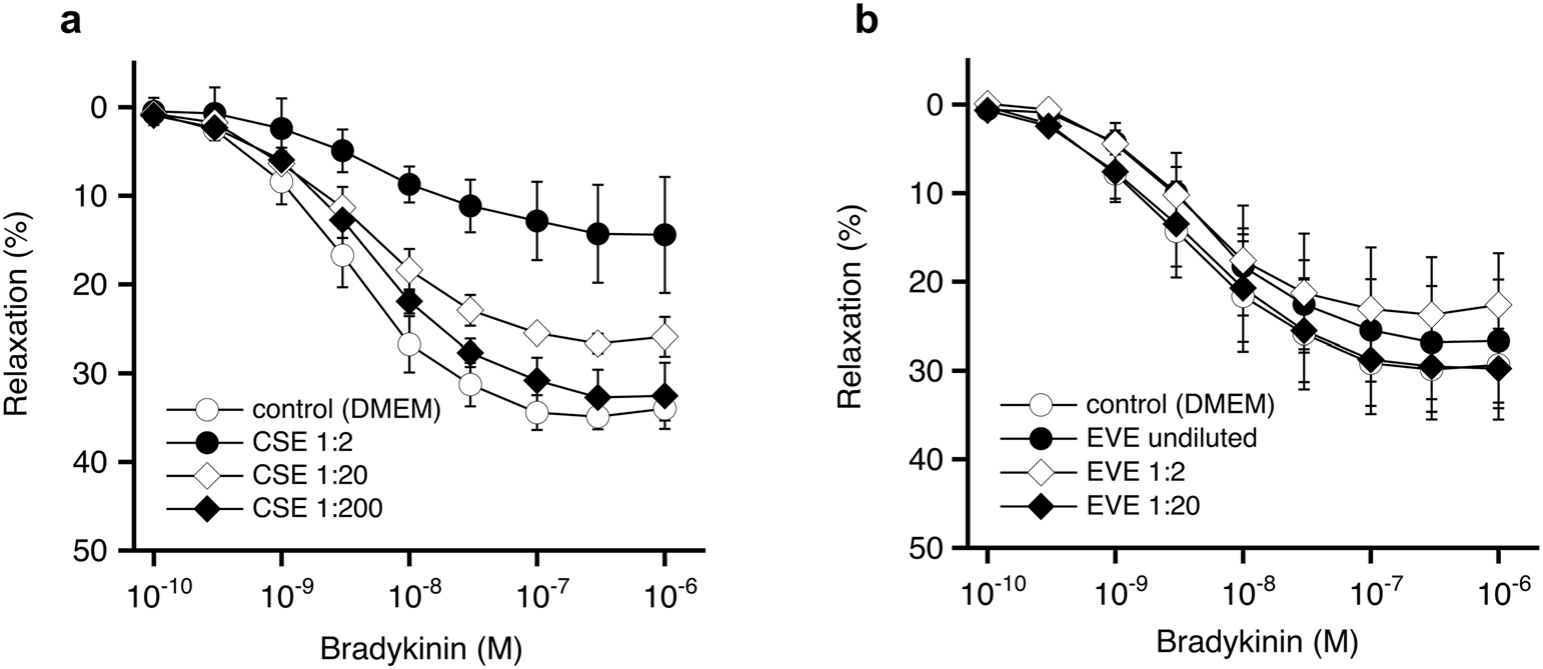
Effects of aerosol extracts on relaxation of porcine coronary arteries to bradykinin. CSE (**a**) impaired endothelial dependent relaxation, whereas EVE (**b**) had no effect. Two rings from a single animal were averaged and counted as an individual experiment. Data are expressed as mean values ± SEM derived from 3 separate hearts. For statistics see Table 3.

**Figure 3:**
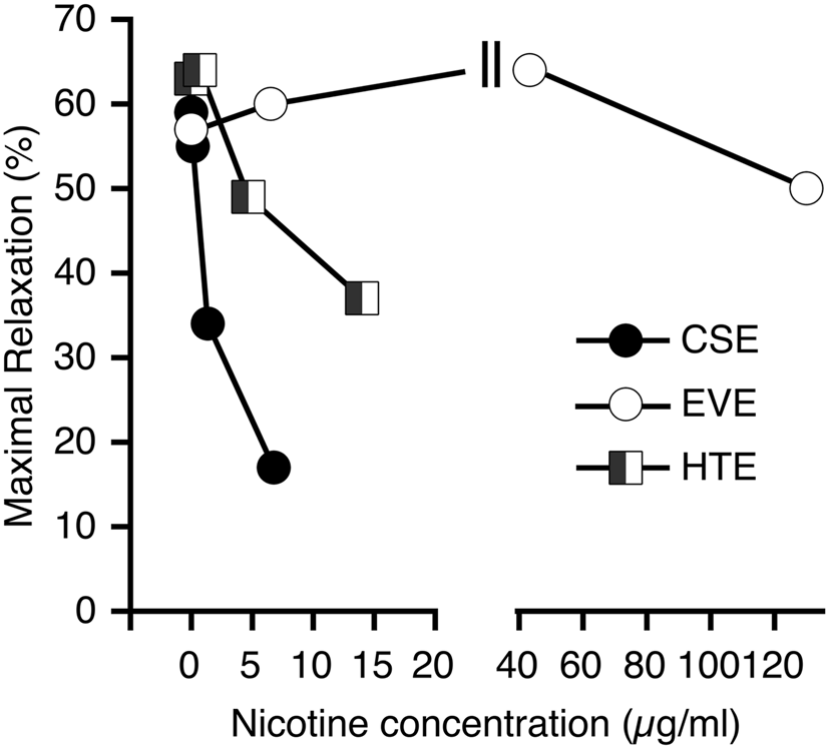
Lack of correlation between the nicotine concentration of the extracts and inhibition of acetylcholine-induced maximal relaxation of rat aortic rings. Data taken from Tab. 1 (nicotine concentration) and Tab. 2 (E_max_ values).

Similar results were obtained with porcine coronary arteries. CSE inhibited relaxation induced by bradykinin, whereas EVE had no significant effect (Fig. 2 and Table 3). The relationship between maximal acetylcholine-induced relaxation and nicotine concentrations of the extracts shown in Fig. 3 demonstrates that the impaired vasodilation observed with CSE and HTE is not caused by nicotine but by other constituents in the aerosols of burned or heated tobacco.

**Table 3:** Effects of CSE and EVE on relaxation of coronary arteries to bradykinin.

Treatment	Nicotine (µg/ml)	E_max_ (%)	EC_50_ (nM)
**CSE**			
Control	–	35 ± 1.3	3.2 (0.7–14.0)
CSE 1:2	8.67	15 ± 6.7*	4.6 (0.1–202.8)
CSE 1:20	1.35	28 ± 2.2	4.4 (0.4–52.5)
CSE 1:200	0.13	33 ± 3.7	4.8 (0.8–30.0)
**EVE**			
Control	–	31 ± 5.6	3.6 (0.4–29.8)
EVE undil	130	28 ± 6.4	5.3 (1.7–16.6)
EVE 1:2	43.3	25 ± 5.2	4.3 (0.7–26.6)
EVE 1:20	6.50	30 ± 4.7	4.5 (0.4–47.6)

Data shown are mean values ± SEM (E_max_) or mean values with 95% confidence interval (EC_50_) from 3 experiments. *p < 0.05 vs. control as determined by ANOVA and Dunnett’s post hoc test.

### Endothelial cell viability

**Figure 4:**
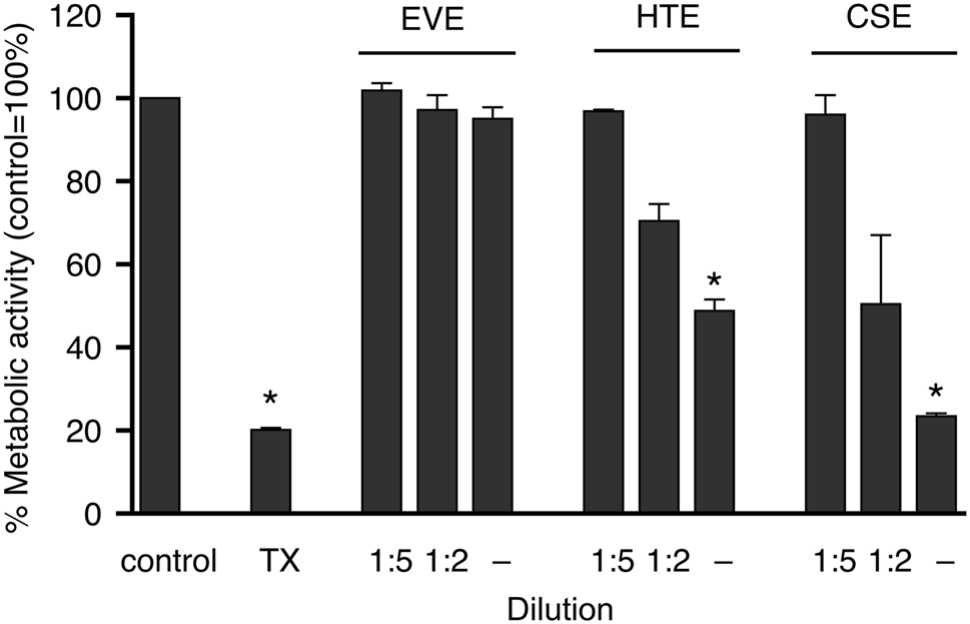
Cytotoxic effects of aerosol extracts on cultured porcine aortic endothelial cells measured by XTT test. While EVE did not affect metabolic activity, even if applied undiluted, CSE and HTE reduced metabolic activity in a concentration-dependent manner. Triton X-100 (1%; TX) served as positive control. Data are expressed as percent of untreated controls and represent mean values ± SEM of 3–8 independent experiments performed in triplicate. *Indicates *p* < 0.05 (ANOVA with Dunnett’s post hoc test).

Potential cytotoxic effects of the extracts on endothelial cells were assessed with an established cell proliferation assay. Figure 4 shows the viability of PAECs which had been exposed for 24 h at 37 °C to aerosol extracts diluted as indicated in the figure. While EVE had no effect even if applied undiluted, CSE and HTE caused concentration-dependent cytotoxicity. Toxicity of undiluted CSE was close to the maximal cytotoxicity observed in the presence of Triton X-100. Since EVE had no effect despite at least tenfold higher nicotine concentration, the results demonstrate that the cytotoxic effects of extracts from burned or heated tobacco are not caused by nicotine.

### Endothelial NO/cGMP signaling

**Figure 5:**
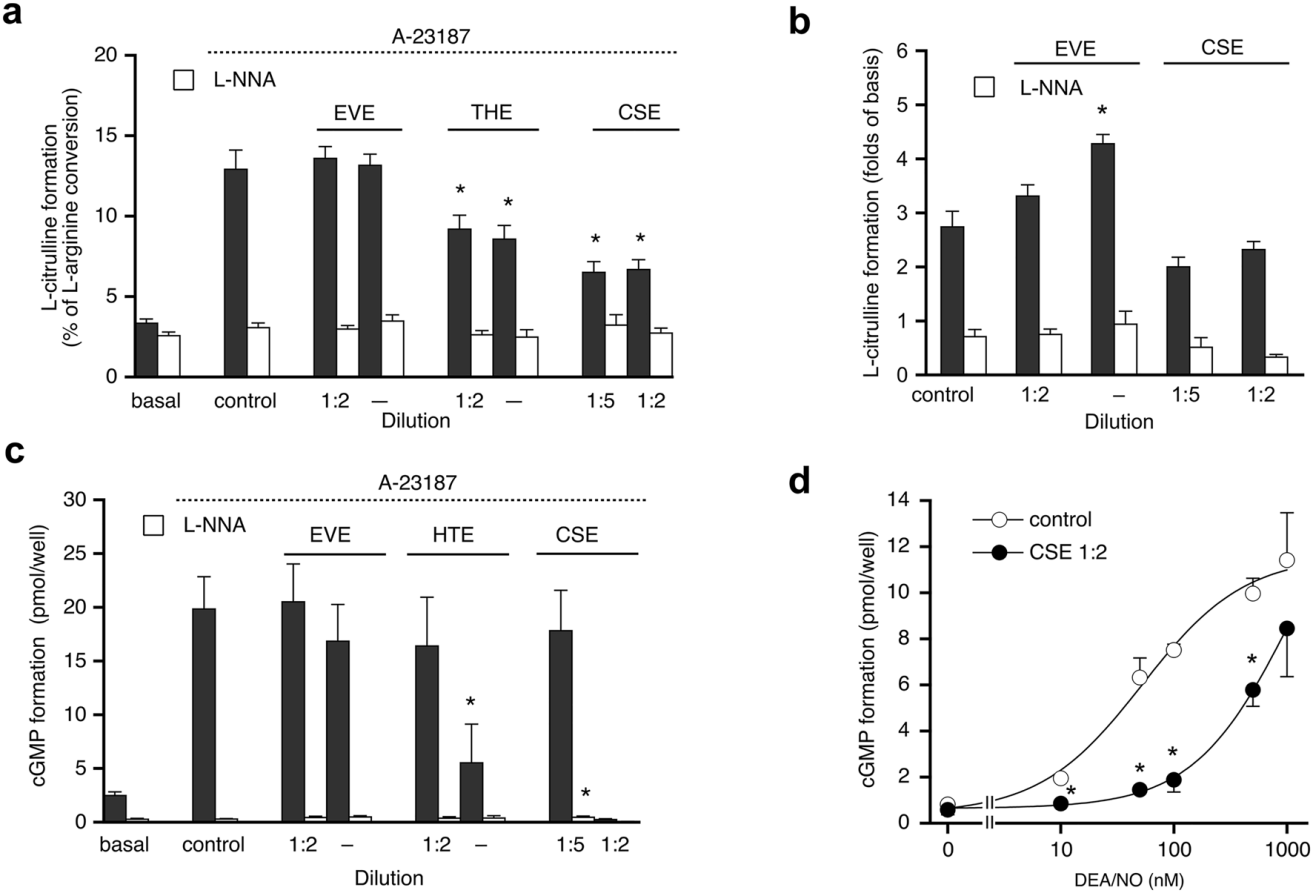
Effects of aerosol extracts on NO/cGMP signaling in PAECs. (**a**) A23187-stimulated eNOS activity (measured as % conversion of L-arginine-to-L-citrulline) was significantly impaired by CSE and HTE after 24 h of preincubation, whereas EVE had no effect. L-NNA (1 mM) was used to control for eNOS-independent L-citrulline formation. Data represent mean values ± SEM of 6 independent experiments performed in triplicate. (**b**) Direct effects on A23187-induced eNOS activity were studied by preincubation of the cells for 15 min in the absence and presence of CSE or EVE. Undiluted EVE significantly increased eNOS activity, while CSE had no effect. Data represent mean values ± SEM of three independent experiments performed in triplicate. (**c**) A23187-stimulated cGMP formation measured by radioimmunoassay was significantly impaired in the presence of HTE and nearly abolished by CSE. Data represent mean values ± SEM of 3–7 independent experiments performed in triplicate. (**d**) Presence of CSE (1:2) caused a pronounced rightward shift of the DEA/NO concentration–response curve. Data represent mean values ± SEM of three independent experiments performed in triplicate. *Indicates *p* < 0.05 (ANOVA with Dunnett’s post hoc test).

To mimic the experimental conditions of the isometric tension studies with isolated aortas, cultured endothelial cells were incubated for 24 h with the extracts, followed by determination of L-arginine-to-L-citrulline conversion and accumulation of cGMP in response to activation of endothelial NO synthase by the Ca^2+^ ionophore A23187. The non-selective NOS inhibitor L-NNA was used to control for basal L-citrulline and cGMP formation independent of eNOS activation by A23187. As shown in Fig. 5, EVE had no effect, whereas CSE and HTE caused various degrees of inhibition of NO/cGMP signaling. CSE and HTE inhibited eNOS activity measured as the formation of L-citrulline by about 30 and 50%, respectively (Fig. 5a). To test for direct effects on eNOS activity we measured L-citrulline formation by the cells incubated for 15 min with EVE or CSE. As shown in Fig. 5b, undiluted EVE significantly increased eNOS activity, while CSE caused a slight, non-significant inhibition of L-citrulline formation. Pure nicotine had no effect (0.2–1.6 mM; n = 3; data not shown), pointing to a non-specific effect of EVE, which was not further studied. The reduction in cGMP formation shown in Fig. 5c was much more pronounced, suggesting additional effects of the tobacco extracts downstream of NO synthesis. This assumption was tested by directly activating endothelial sGC with the NO donor DEA/NO. Figure 5d shows that 1:2 diluted CSE markedly inhibited cGMP formation in the presence of up to 0.3 µM DEA/NO, while inhibition was much less pronounced at higher concentrations of the NO donor. CSE increased the EC_50_ value of DEA/NO from 52 ± 15 to 447 ± 37 nM (calculated by forcing the curve through the maximum of the control). The observation that the degree of inhibition decreased with increasing NO concentrations most likely reflects the limited NO scavenging capacity of the extract (see Fig. 7a below). The results may partially explain the pronounced inhibition of A23187-induced cGMP accumulation shown in Fig. 5c.

### Effects of CSE and EVE on the activity of purified sGC

**Figure 6:**
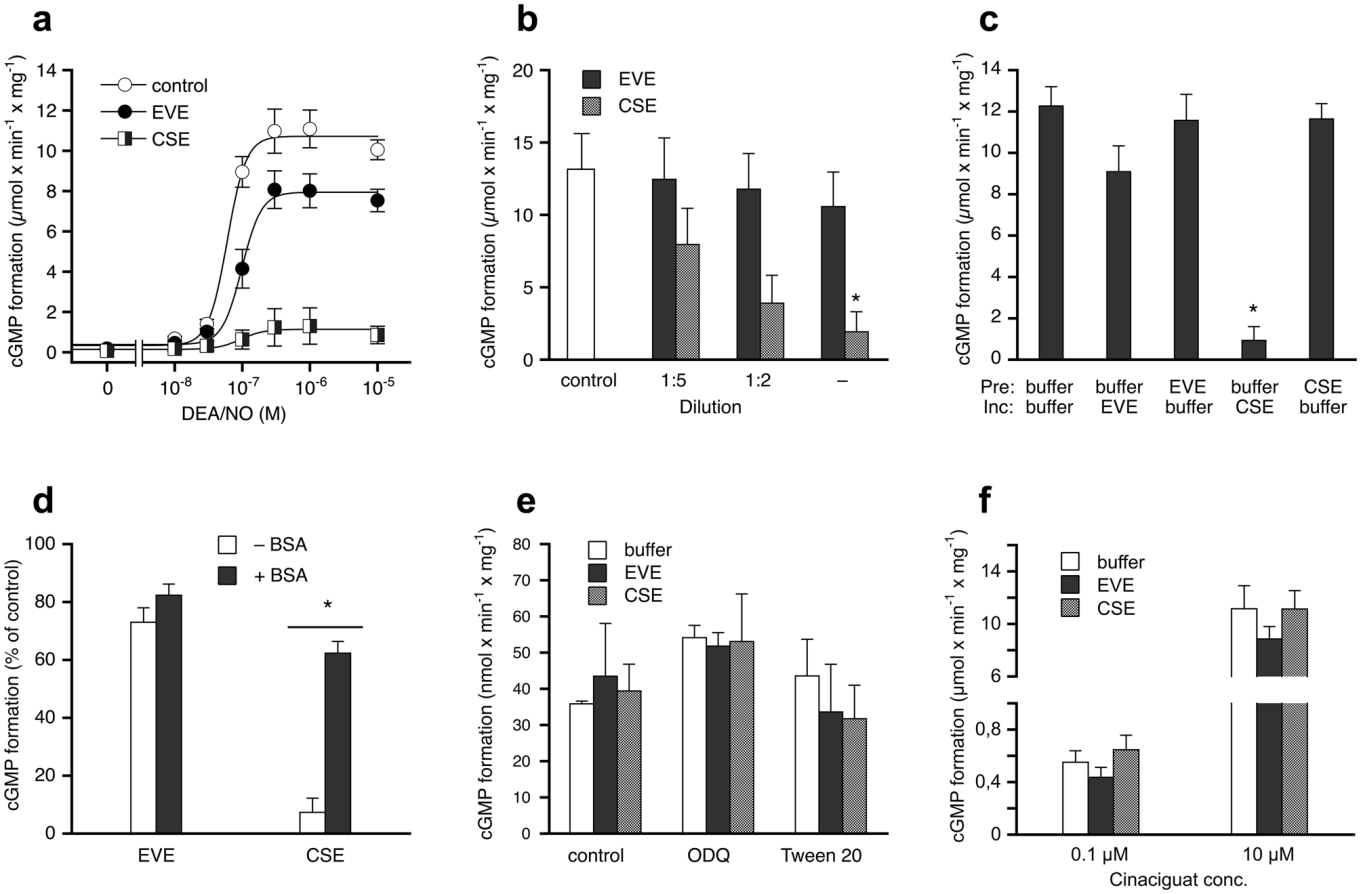
Effects of aerosol extracts on purified sGC. The formation of cGMP was measured in the absence or presence of the extracts. (**a**) CSE caused almost complete inhibition of DEA/NO-stimulated enzyme activity, whereas the effect of EVE was much less pronounced. (**b**) The inhibition caused by CSE shown in panel (**a**) was concentration-dependent. (**c**) Preincubation experiments revealed that inhibition of sGC by the extracts was fully reversed by dilution of the CSE-exposed enzyme. (**d**) The presence of BSA (0.5 mg/ml) prevented the inhibition caused by CSE. (**e**) Basal enzyme activity (measured in the absence of DEA/NO) was not affected by the extracts (control). In addition, the extracts had no significant effects on the activity of the enzyme oxidized with ODQ or heme-depleted with Tween 20. (**f**) The extracts did not affect cGMP formation by Tween 20-treated sGC in the presence of the NO-independent heme-site activator cinaciguat. Data are expressed as mean values ± SEM derived from 3 independent experiments. *Indicates *p* < 0.05 in paired Student’s t-test (**d**), or ANOVA with Dunnett’s post hoc test *vs.* control (**b**) or *vs.* buffer/buffer control (**c**), respectively.

The effects of CSE and EVE on sGC activity were studied with the purified enzyme. Figure 6a shows that undiluted EVE slightly reduced enzymatic cGMP formation in the presence of increasing concentrations of DEA/NO, but neither the reduction of v_max_ (from 11.1 ± 1.1 to 8.5 ± 1.0 µmol × min^−1^ × mg^−1^) nor the increase of the EC_50_ value (from 63.1 ± 2.5 to 107.3 ± 16 nM) were statistically significant (ANOVA with Dunnett’s post hoc test). In contrast, undiluted CSE almost completely inhibited sGC stimulation by DEA/NO, resulting in a reduction of v_max_ 1.4 ± 0.9 µmol × min^−1^ × mg^−1^ (~ 12% of control) and an increase of EC_50_ to 145.5 ± 30.4 nM (2.3-fold vs. control). The effect of CSE was concentration-dependent (Fig. 6b), fully reversible (Fig. 6c), and almost completely prevented in the presence of 0.5 mg/ml BSA (Fig. 6d).

Basal sGC activity measured in the absence of DEA/NO was not affected by any of the three extracts. Similarly, the extracts had no effects on the activity of heme-free or oxidized sGC, measured in the presence of Tween 20 and ODQ, respectively (Fig. 6e), or the cinaciguat-activated enzyme (Fig. 6f). Taken together, these data suggest that CSE interferes with the NO stimulation of sGC.

### Correlation of sGC activity with NO release from DEA/NO

**Figure 7:**
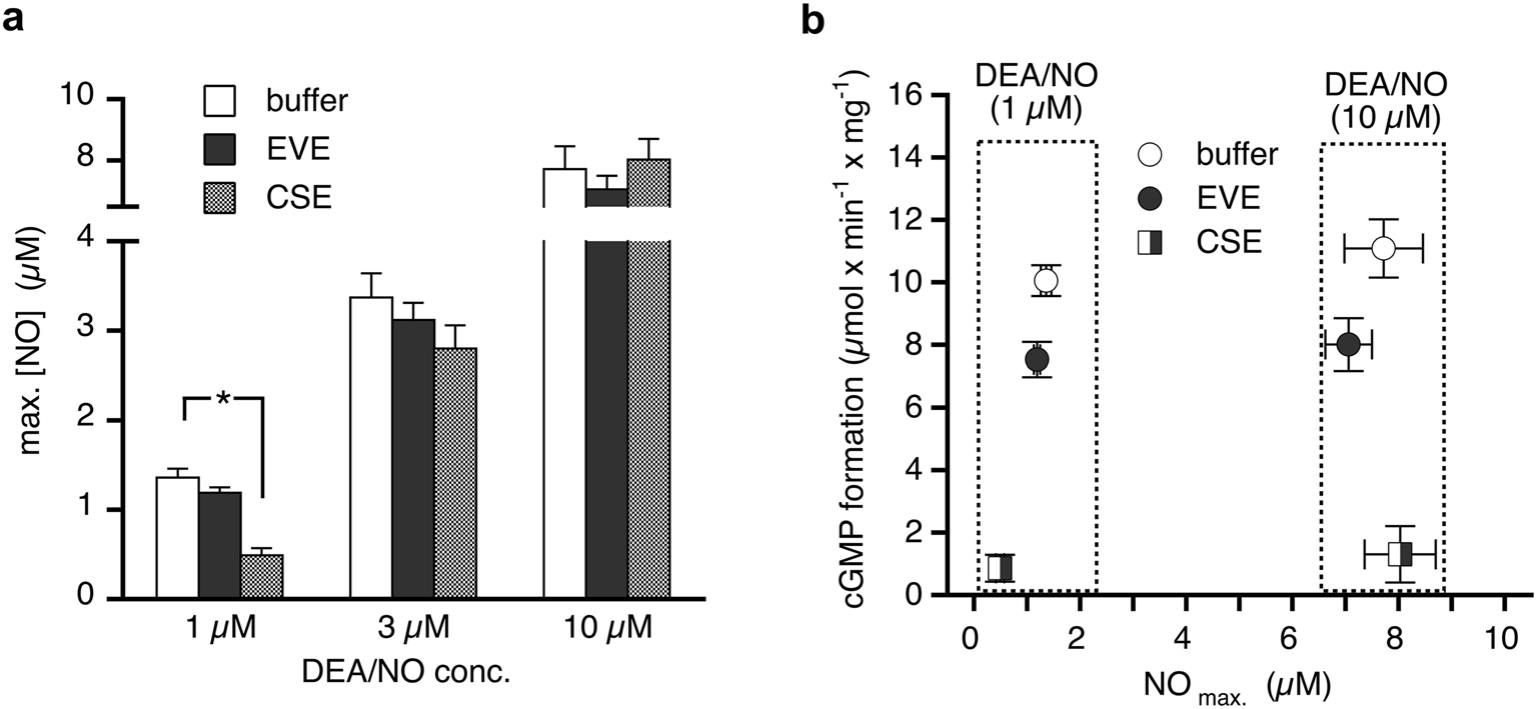
Effects of aerosol extracts on NO release from DEA/NO. Peak concentrations of released NO were measured with a Clark-type electrode in the absence or presence of EVE and CSE. (**a**) CSE significantly reduced maximal NO concentration released from 1 µM DEA/NO, whereas EVE had no effect. Neither of the extracts affected NO release from 3 and 10 µM DEA/NO. (**b**) The correlation of sGC activity with measured NO peak concentrations under identical conditions shows that CSE caused pronounced inhibition of NO-stimulated sGC beyond NO scavenging. Data are expressed as mean values ± SEM from three independent experiments. *Indicates *p* < 0.05 in ANOVA with Dunnett’s post hoc test.

Figure 7a shows the peak concentrations of NO released from 1, 3, and 10 µM DEA/NO in the absence and presence of EVE and CSE. The extracts had no detectable effects at high DEA/NO concentrations (3 and 10 µM), but NO released from 1 µM DEA/NO was significantly scavenged by CSE (from 1.36 ± 0.1 to 0.49 ± 0.08 µM, corresponding to 36% of control). Scavenging was reduced but not prevented in the presence of 0.5 mg/ml BSA (63% of control). Limited electrode sensitivity precluded reliable NO release measurements from lower DEA/NO concentrations. The correlation of sGC activity with NO release measured under identical conditions (Fig. 7b) illustrates the pronounced inhibition of NO-stimulated sGC by CSE.

## Discussion

The present study unequivocally demonstrates that the inhibition of agonist-induced endothelium-dependent relaxation, which has been consistently observed with inhaled tobacco smoke^3^, is not mediated by nicotine. CSE significantly inhibited the relaxation of aortas and coronary arteries to acetylcholine and bradykinin, respectively, whereas EVE had no effect despite much higher nicotine concentrations. The finding that CSE did not affect relaxation induced by DEA/NO indicates that tobacco smoke constituents specifically impair endothelium-dependent vasodilation. Furthermore, pronounced inhibition was observed with CSE diluted to a concentration tenfold lower than the threshold of cytotoxicity (1:20 *vs.* 1:2), suggesting interference with agonist-induced NO/cGMP signaling in endothelial cells rather than a non-specific toxic effect.

The observation that CSE impaired agonist-induced relaxation without affecting the response to DEA/NO is in apparent conflict with the results obtained with cultured endothelial cells and purified sGC. The activity of eNOS, measured as L-arginine-to-L-citrulline conversion in response to stimulation of the cells with A23187, was inhibited by about 50% in the presence of CSE diluted 1:2, while the extract caused virtually complete inhibition of vascular relaxation at 1:3 dilution (cf. Figures 1a and 5a). When we tested the acute effects of the extract on endothelial cells, we observed a similar tendency of inhibition of L-citrulline formation, but the effects were not statistically significant (see Fig. 5b).

Endothelial cGMP accumulation in response to A23187 or low concentrations of DEA/NO (up to 30 nM) was abolished by CSE at 1:2 dilution (see Fig. 5, panels c and d). Similarly, the activity of DEA/NO-stimulated sGC was inhibited in a concentration-dependent manner by CSE (see Fig. 6b). In comparison, the extract had no significant effect on DEA/NO-induced vasodilation (Fig. 1b). Since basal sGC activity (measured in the absence of added DEA/NO) was not inhibited by CSE and the extract had no effect on the cinaciguat-stimulated or heme-free enzyme (see Fig. 6, panels e and f), the extract apparently interferes with the stimulation of sGC by NO. The protective effect of BSA (see Fig. 6d) suggests that CSE causes reversible oxidative modification of the enzyme, but NO scavenging (Fig. 7) may additionally contribute to the observed effect. Taken together, these data suggest that inhibition of sGC by constituents of tobacco smoke results in impaired NO stimulation in endothelial cells, while the enzyme is protected from this modification in smooth muscle cells by a protein acting like BSA, which was used as an experimental model protein to protect sGC.

The most relevant information on the effects of e-cigarette vapor on endothelium-dependent vasodilation is provided by in vivo measurements of FMD upon acute inhalation or chronic use of aerosols generated by tobacco cigarettes, e-cigarettes, or THS. There is conclusive evidence that FMD is significantly reduced in chronic smokers^7,51,52^. Improvement of FMD by supplementation with antioxidants^7,53^ indicates that the endothelial dysfunction of smokers may be caused by vascular oxidative stress, resulting in reduced bioavailability of endothelium-derived NO. The widespread use of e-cigarettes as alternative nicotine sources has raised the question of whether nicotine contributes to this effect. The rapid reversal of impaired FMD in smokers switching to nicotine-containing e-cigarettes^24,25^ and identical FMD of e-cigarette users and non-users^26^ appears to exclude nicotine causing chronic damage to the endothelium, but several studies reported an acute reduction of FMD after short-term e-cigarette use^17–20^. The issue is further complicated by some reports showing that inhaled e-cigarette vapor caused an acute reduction of FMD even in the absence of nicotine^20,54,55^. These findings may reflect vascular oxidative stress caused by aerosol inhalation. However, while it is conceivable that inhaled propylene glycol, glycerol, or flavorings affect the function of lung epithelial cells, effects on blood vessels are not particularly plausible. Recently, it was reported that the inhalation of inert carbon nanoparticles impaired FMD to a similar degree as aerosols from cigarettes, e-cigarettes, or HTS^56^. As bilateral cervical vagotomy blocked this effect in rats, airway irritation appears to trigger vagus nerve signaling, resulting in endothelial dysfunction. The authors conclude that there is no single constituent or class of constituents responsible for acute impairment of endothelial function caused by aerosol inhalation^56^. These findings may at least partially explain the divergent results on acute *vs.* long-term effects of aerosol inhalation and agree well with our conclusion that the impaired vasodilation caused by tobacco smoke is not mediated by nicotine.

The effects of HTE were between those of EVE and CSE concerning impaired agonist-induced vasodilation, inhibition of eNOS activity, endothelial cGMP accumulation, and cytotoxicity. Our results agree well with studies comparing the emissions and health risks of e-cigarettes and THS (for a comprehensive review, see Murkett et al.^57^), suggesting that heating tobacco results in partial extraction of toxic compounds, which mediate the observed effects on endothelial NO/cGMP signaling. Some studies reported that aerosols from heated tobacco cause endothelial dysfunction, measured as FMD, to a similar degree as tobacco smoke in acutely exposed rats^58^ and chronic smokers^59,60^. We observed a significant inhibition of acetylcholine-induced relaxation of rat aortic rings. However, the effect was less pronounced than that of CSE despite much higher nicotine concentrations (see Table 2). Figure 3 illustrates that the nicotine concentration of the extracts does not correlate with maximal inhibition of vasorelaxation, further confirming that the effect is not caused by nicotine.

### Limitations of the study

We tested extracts prepared from the different aerosols, but the properties of extracts do not necessarily reflect the properties of the aerosols that users inhale. In addition, it should be considered that we used only one type of vaporizer under defined conditions. Extracts from aerosols generated with other devices operating at different power levels could exert distinct biological effects.

Another limitation is the focus on endothelium-dependent relaxation and NO/cGMP signaling. Since impaired vasodilation is only one of several hallmarks of endothelial dysfunction, our results do not exclude other detrimental effects of inhaled nicotine on endothelial cell function. Besides its role in vasodilation, the endothelium inhibits platelet adhesion and aggregation, regulates inflammatory processes, is essential for capillary integrity, and promotes angiogenesis through cell migration. There is general agreement that the inhalation of tobacco smoke negatively affects these biological processes^3^, but the role of nicotine is controversial concerning angiogenesis/cell migration^61–63^, endothelial barrier function^64,65^, and platelet activation^66–68^. There is conclusive evidence that inhalation of e-cigarette vapor increases markers of oxidative stress and inflammation^19,20^, but e-cigarettes induce substantially less pronounced biological responses than tobacco smoke^69^, and a recent cross-sectional study found no differences in the levels of inflammatory markers between e-cigarette users and non-users^26^. Nevertheless, we wish to emphasize that e-cigarette vapor contains potentially toxic compounds besides nicotine, which may affect the function of vascular endothelial cells if efficiently resorbed into the systemic circulation. Further work is warranted to clarify this issue.

## Data Availability

The original data generated in this study will be made freely available in the Zenodo repository (https://zenodo.org/deposit/7963137).
